# EGR1 promoted anticancer effects of Scutellarin via regulating LINC00857/miR‐150‐5p/c‐Myc in osteosarcoma

**DOI:** 10.1111/jcmm.16809

**Published:** 2021-08-04

**Authors:** Jian Han, Peng Wang, Xin Xia, Li Zhang, He Zhang, Yu Huang, Xiaodong Li, Wenzhi Zhao, Lu Zhang

**Affiliations:** ^1^ The Second Affiliated Hospital Institute of Cancer Stem Cell Dalian Medical University Dalian China; ^2^ Laboratory of Pathogenic Biology College of Basic Medical Science Dalian Medical University Dalian China; ^3^ Department of Pathophysiology College of Basic Medical Sciences Dalian Medical University Dalian China

**Keywords:** EGR1, LncRNA, osteosarcoma, scutellarin

## Abstract

Scutellarin, an active flavone extracted from *Erigeron breviscapus*, is known to exhibit antitumour activity in many cancers. However, the effects of Scutellarin on osteosarcoma remain unclear. In this study, we found that Scutellarin suppressed osteosarcoma cell growth, induced cell apoptosis and inhibited tumorigenesis. Mechanistically, our data revealed that EGR1 was significantly increased under Scutellarin treatment. Increased EGR1 enhanced tumour‐suppressive effects of Scutellarin on osteosarcoma cells via transcriptionally downregulating LINC00857 expression. Additionally, we found that LINC00857 acted as a competitive endogenous RNA of miR‐150‐5p and inhibited the activity of miR‐150‐5p, which resulted in c‐Myc increase. Scutellarin could suppress c‐Myc protein levels through decreasing LINC00857 expression in osteosarcoma. Thus, these findings demonstrate that EGR1/ LINC00857/miR‐150‐5p/c‐Myc axis plays a key role in promoting anticancer effects of Scutellarin and Scutellarin might have potential clinical implication in osteosarcoma clinical treatment.

## 1 INTRODUCTION

Osteosarcoma (OS) is one of the most common malignant bone tumours that seriously endangers the health of children and adolescents. Although many advanced therapeutic strategies for the treatment of osteosarcoma were described, the survival rate has not been improved owing to metastasis and resistance to conventional chemotherapies that led to tumour recurrence.^1^, ^2^ Thus, it is of great significance to uncover new effective agents and develop new therapeutic strategies with high safety to improve this adverse consequence of patients with osteosarcoma.

Scutellarin extracted from the traditional Chinese herb *Erigeron breviscapus* and *Asarum*.^3^ Accumulating evidence indicates that Scutellarin is generally used in therapy of cardiovascular diseases for many years because of its vasodilation, anti‐inflammation, antioxidation, anticoagulation and antithrombotic activities.^4^ Recently, Scutellarin was reported to be an antitumour factor to promote cell apoptosis and inhibit cell growth, invasion and migration in many human tumours including hepatocellular carcinoma, tongue squamous carcinoma, renal cancer and lung cancer,^5^, ^6^, ^7^, ^8^ but the role of Scutellarin in OS cells has been poorly understood.

Early growth response gene‐1 (EGR1) is an immediate early gene, which owns a DNA‐binding domain composed of three zinc‐finger motifs and appears to activate transcription by binding to DNA as a monomer.^9^ EGR1 can be expressed rapidly under the induction of various stimuli such as growth factors, hormones and neurotransmitters, and then regulate downstream target genes.^10^ Emerging data suggest that EGR1is dysregulation in several human cancers and plays an essential role in tumorigenesis.^11^ Notably, the antitumour mechanism of many natural compounds is closely related to the expression of EGR1. For example, Coptis chinensis induced apoptosis of HCC cells by promoting EGR1‐induced NAG‐1 promoter activity.^12^ However, the role of EGR1 in the treatment of osteosarcoma by Scutellarin is still unknown.

Long non‐coding RNAs (lncRNAs) are longer than 200 nucleotides RNAs, which have no protein‐coding capacity.^13^ Many reports have shown that lncRNAs play vital regulatory roles in many human diseases, especially in malignant tumours.^14^ LINC00857 (long intergenic non‐protein‐coding RNA 857) has been reported to play a carcinogenic factor in several human cancers and regulated cancer cell growth, death and metastasis. For example, LINC00857 was increased in hepatoma cell lines and depletion of LINC00857 inhibited the progression of hepatocellular carcinoma.^15^ Although several studies have suggest that LINC00857 play a key role in cancer, its functions in response to Scutellarin remain unclear.

Here, we found the potential of Scutellarin to inhibit growth of osteosarcoma cells at the cellular level and in animal models. Mechanistically, we uncovered that Scutellarin induced EGR1 increase in osteosarcoma cells, which led to cell apoptosis increase and cell growth decrease. Subsequently, our data indicated that LINC00857 was decreased under Scutellarin treatment and contributed to Scutellarin‐induced c‐Myc downregulation via acting as a miR‐150‐5p sponge. Except that, we also found that EGR1 bound to the region of LINC00857 promoter, suppressing LINC00857 expression under Scutellarin treatment. Taken together, these results suggest that EGR1/Linc00857/miR‐105‐5p/c‐Myc signal axis plays an essential role in antitumour effects of Scutellarin on osteosarcoma.

## 2 MATERIALS AND METHODS

### 2.1 Reagents and antibodies

We purchased Scutellarin from Meilune company (Cat: MB7004‐S). It was dissolved in Dimethyl Sulfoxide to confect primary solution with final concentration of 18.5g/L (40 mmol/L) for in vitro experiments. These following antibodies were used: EGR1 (Proteintech, 22008‐1‐AP), GAPDH (Santa Cruz Biotechnology, SC‐25778) and c‐Myc (Cell Signalling Technology, 18583S).

### 2.2 Cell culture and transfection

The human osteosarcoma cancer cell lines 143B and U2OS were provided by the American Type Culture Collection. The cells were cultured in Dulbecco’s modified Eagle’s medium containing 10% foetal bovine serum and streptomycin (100 μg/ml) and were cultured at 37°C in a humidified atmosphere with 5% CO_2_. The siRNA for LINC00857, EGR1, c‐Myc and miR‐105‐5p mimics, and inhibitors were ordered from GenePharma. For plasmid transfection, Lipofectamine 3000 (Invitrogen) was used in accordance with manufacturer’s instructions.

### 2.3 MTT and Hoechst 33342 staining assay

Briefly, we seeded osteosarcoma cells into 96‐well plates at the density of 7 × 10^4^ cells per well. After 48 h of treatment, 20 μl of MTT reagent (5 mg/ml) was added immediately to each well and incubated for another 4 h. Then, we removed the culture medium and added 200 μl dimethyl sulfoxide to each well. At the end, absorbance value at 570 and 630 nm was assessed by using microplate reader (PerkinElmer).

For Hoechst 33342 staining assay, osteosarcoma cells were cultured in six‐well plates and treated with or without Scutellarin for 48 h. Then, these cells were stained with Hoechst 333342 solution (Sigma) and incubated for 30 min. After washing with PBS for three times, the morphologic changes of cells were recorded by fluorescence microscope (Olympus).

### 2.4 Colony formation assay

143B and U2OS cells were collected and prepared into single‐cell suspension in complete culture medium. After cell counting, 143B and U2OS cells were cultured in six‐well plates with 200 or 400 cells per well. After 24 h, Scutellarin was added to each well. These cells were cultured for 2 weeks and then were stained by 0.04% crystal violet, and proliferation ability was assessed by taking pictures of the above cells.

### 2.5 Annexin V‐FITC staining and flow cytometry

The protocol was provided by the manufacturer, and its instructions were followed (Yeasen). In brief, the cells were pretreated with Scutellarin, harvested by centrifugation at 300 *g* in 4°C and suspended in 100 μl binding buffer. Then, 5 μl Annexin V‐FITC and 10 μl propidium iodide (PI) staining solution were added for a 10‐min incubation at room temperature in darkness. Then, the cells were suspended with 400 μl binding buffer and mixed gently. The flow cytometry (BD Biosciences) analysis was conducted to test cell apoptotic events.

### 2.6 Animal experiments

Animal experiments were conducted according with the National Institute of Health Guide for the Care and Use of Laboratory Animals with the approval of the Animal Research Committee of Dalian Medical University. 143B cells were subcutaneously injected into the nude mice. After 7 days, the mice were randomly divided into two groups. The control group was treated with 100 μl PBS by intragastric administration. The test group was treated with 100 μl SCU (60 mg/kg) diluted by PBS through the same administration every day. All mice need to be observed every 2 days, and the tumour volume was measured. The 143B tumour volume was calculated using the formula *V* = 1/2 (length × width^2^). After 12 days of administration of Scutellarin or normal PBS, the mice were killed via cervical dislocation, and then, the tumours were taken pictures, measured weights and fixed for further experiments after carefully removed.

### 2.7 Real‐time RT‐PCR and RT‐PCR

Total cellular RNA was isolated by using Trizol (Invitrogen). According to the manufacturer’s instructions, PrimeScriptTM RT kit (Takara, RR047A) was used to synthesize cDNA. The primers for EGR1 and actin were as follows: EGR1: F:5‐TGACCGCAGAGTCTTTTCCT‐3 and R: 5‐TGGGTTGGTCATGCTCACTA‐3; Actin: F: 5‐GACCTGACTGACTACCTCATGAAGAT‐3 and R: 5 ‐GTCACACTTCATGATGGAGTTGAAGG‐3.

### 2.8 Dual‐luciferase reporter assay and ChIP assay

The dual‐luciferase reporter assay and ChIP were performed as previously described.^16^

### 2.9 Statistics and data analyses

The data were expressed as the means ± SD, and the statistical evaluation was performed using one‐way analysis of variance (ANOVA). Values of *p* < .5 were considered statistically significant.

## 3 RESULTS

### 3.1 Scutellarin decreases osteosarcoma cell growth and increases cell apoptosis

**FIGURE 1:**
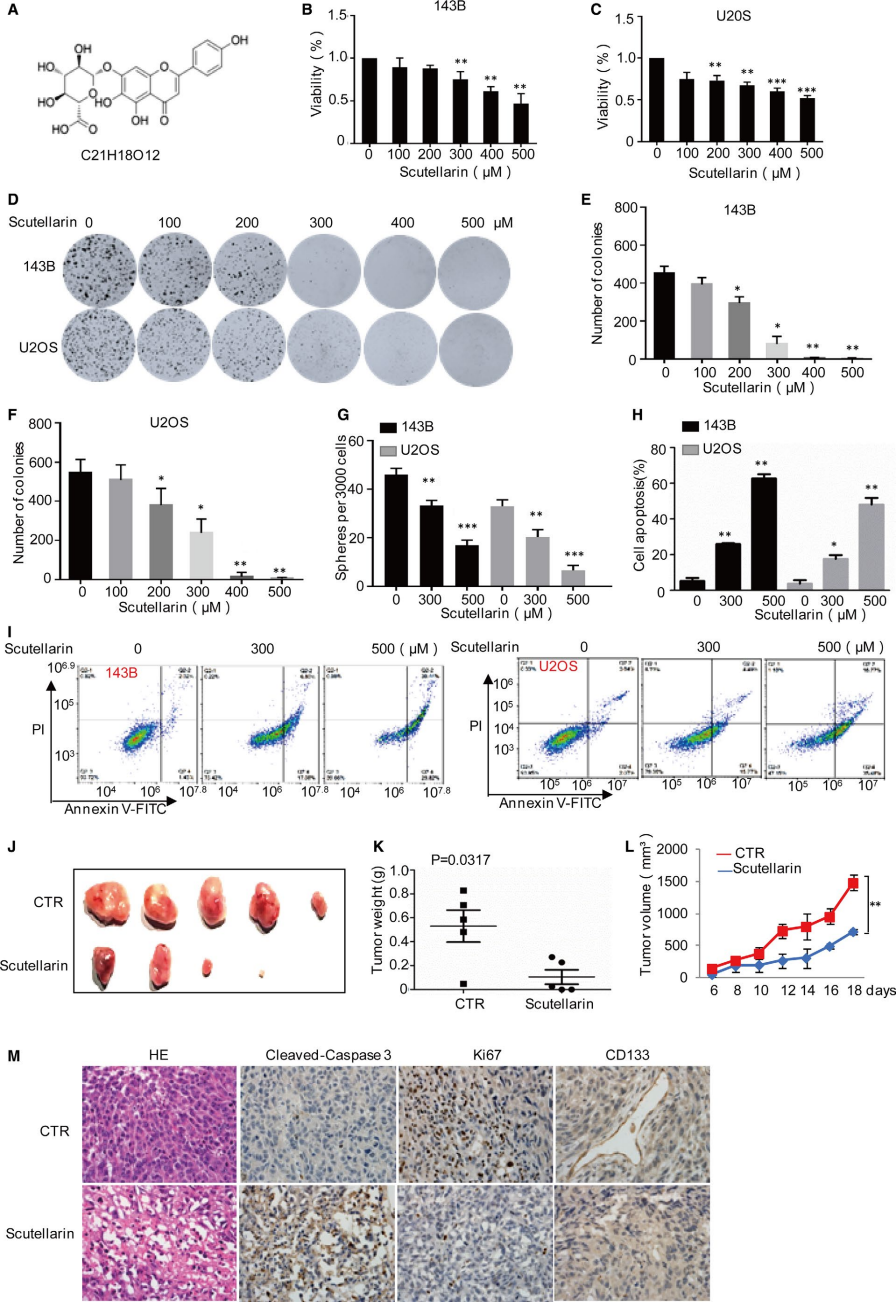
Scutellarin decreases osteosarcoma cell growth and induces cell apoptosis. (A) Chemical structure of Scutellarin. (B,C) 143B and U2OS cells were treated with or without Scutellarin for 48 h, and cell viability was detected by MTT assay. (D‐F) 143B and U2OS cells were treated with Scutellarin as indicated. Cell growth was analysed by colony formation assay. (G) The mammosphere‐forming abilities were analysed by sphere formation assays. (H,I) 143B and U2OS cells were treated with or without Scutellarin for 48 h as indicated. Cell apoptosis was detected by flow cytometer. (J‐L) 143B cells were subcutaneously injected into nude mice (*n *= 5 in each group) for tumour formation. After Scutellarin treatment for 12 days, mice were humanely killed. Tumour volumes were monitored during the detectable period in nude mice. Tumour weights were measured after xenograft was harvested. (M) The xenograft tumours treated by Scutellarin were analysed by H&E staining, IHC staining using ki67, cleaved caspase 3 and CD133. For (B), (C), (E), (F), (G), (H) and (L), the results represent the mean ± SD of three independent experiments, *p < .05, **p < .01, ***p < .001 vs. control

The major chemical structure of Scutellarin is shown in Figure 1A. To investigate anticancer effects of Scutellarin on osteosarcoma cells, a series of assays were used to examine the influences of Scutellarin on cell viability and growth in 143B and U2OS cells. Our data indicated that Scutellarin significantly decreased cell viability and inhibited cell proliferation of all two osteosarcoma cell lines in a dose‐dependent manner (Figure 1B‐F). Then, we carried out mammosphere formation to assess the effects of Scutellarin on osteosarcoma stem cells and found that Scutellarin dramatically decreased the sphere formation efficiency (Figure 1G).

In the following flow cytometric analyses of cell apoptosis, we found that treatment with Scutellarin led to a significant increase in apoptotic numbers of osteosarcoma cells (Figure 1H‐I). To further determine the influence of Scutellarin on tumour formation ability of osteosarcoma cells in vivo, we generated osteosarcoma xenograft nude mouse models using 143B cell line and randomly divided the nude mice into two groups to receive control saline and Scutellarin by daily oral gavage. Compared with the control saline group, Scutellarin significantly decreased the tumour volume and weight (Figure 1J‐L). Subsequently, the immunohistochemistry analysis assay was performed to detect the resected 143B xenograft tumour tissues from the control and treatment groups using the Ki‐67, cleaved caspase 3 and CD133 antibodies. Ki‐67, cleaved caspase 3 and CD133 expression were detected to examine the proliferative apoptotic status and angiogenesis of different tumour samples. As shown in Figure 1M, Scutellarin treatment downregulated the percentages of Ki‐67‐positive cells, upregulated the proportions of cleaved caspase 3–positive cells and decreased CD133 expression. Collectively, these results indicate that Scutellarin can prevent the growth and induce the apoptosis of osteosarcoma cells in vivo and in vitro.

### 3.2 Scutellarin upregulated EGR1 expression in osteosarcoma cells

**FIGURE 2:**
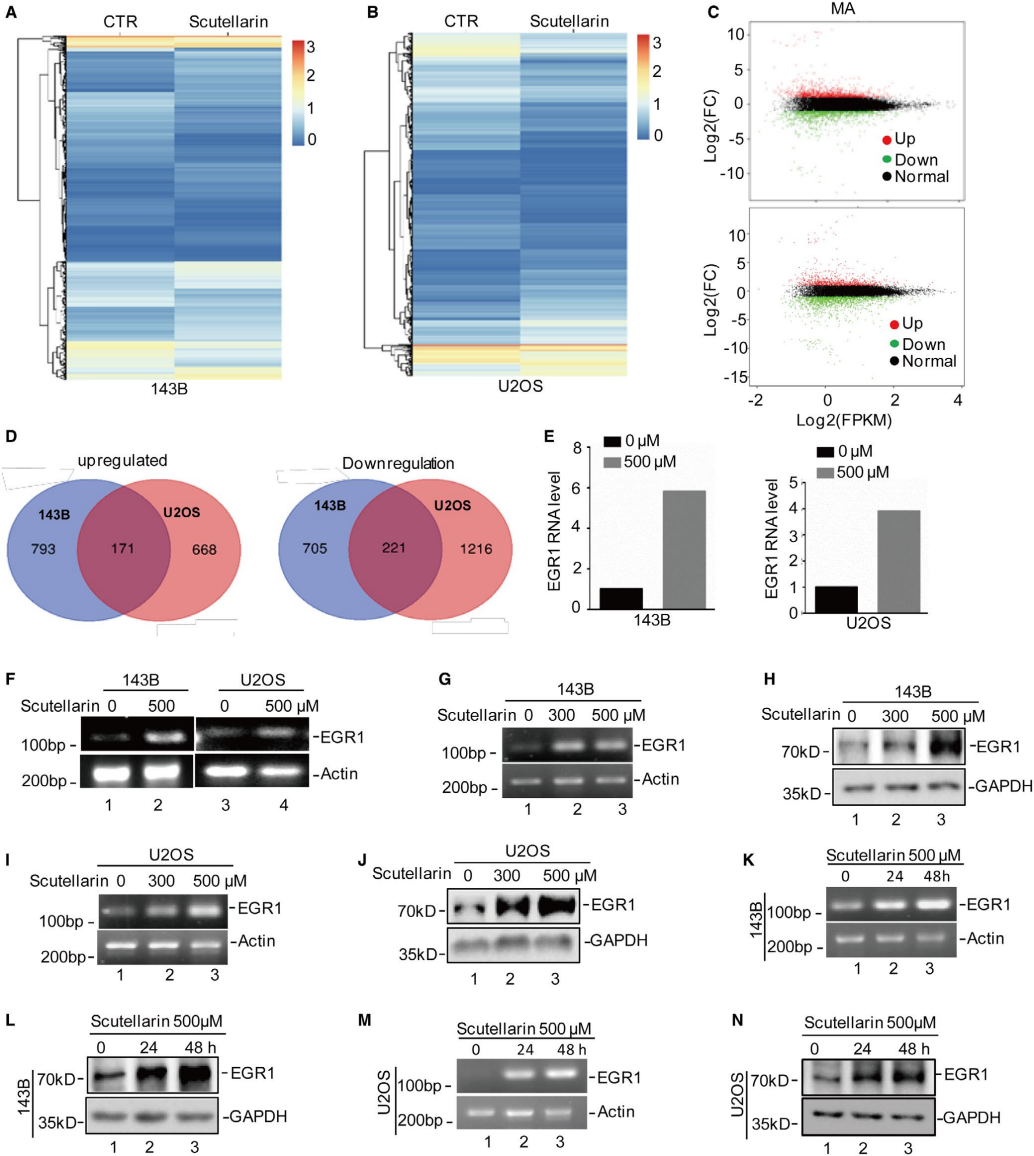
Scutellarin upregulates EGR1 expression in osteosarcoma cells. (A,B) 143B and U2OS cells were treated with or without 500 μM Scutellarin for 48 h. Then, the cells were subjected to RNA sequencing analysis. (C,D) The upregulated and downregulated genes were screened in 143B and U2OS cells. (E) The EGR1 expression was listed. (F‐N) 143B and U2OS cells were treated with Scutellarin as indicated concentrations and times. EGR1 expression was measured by RT‐PCR and Western blotting

To uncover the antitumour mechanism of Scutellarin, osteosarcoma cells were treated with or without 500 μM Scutellarin for 48 h. The mRNA profiles were investigated by RNA sequencing analysis. As shown in Figure 2A‐C, we found that 1498 (793 upregulated and 705 downregulated) and 1884 (668 upregulated and 1216 downregulated) differentially expressed genes in 143B and U2OS cells under Scutellarin treatment. Not surprisingly, many altered genes (171 upregulated and 221 downregulated) exhibited overlapping expression in U2OS and 143B cells (Figure 2D). Among the 171 genes, EGR1 was significantly upregulated under Scutellarin treatment (Figure 2E).

To further confirm it, osteosarcoma cells were treated with Scutellarin using different concentrations and times. EGR1 expression was measured by RT‐PCR and Western blotting. Consistent with RNA sequencing analysis, Scutellarin significantly increased the EGR1 expression (Figure 2F‐N). Taken together, these results suggest that EGR1 is increased under Scutellarin treatment in osteosarcoma.

### 3.3 EGR1 contributes to the antitumour effects of Scutellarin in osteosarcoma cells

**FIGURE 3:**
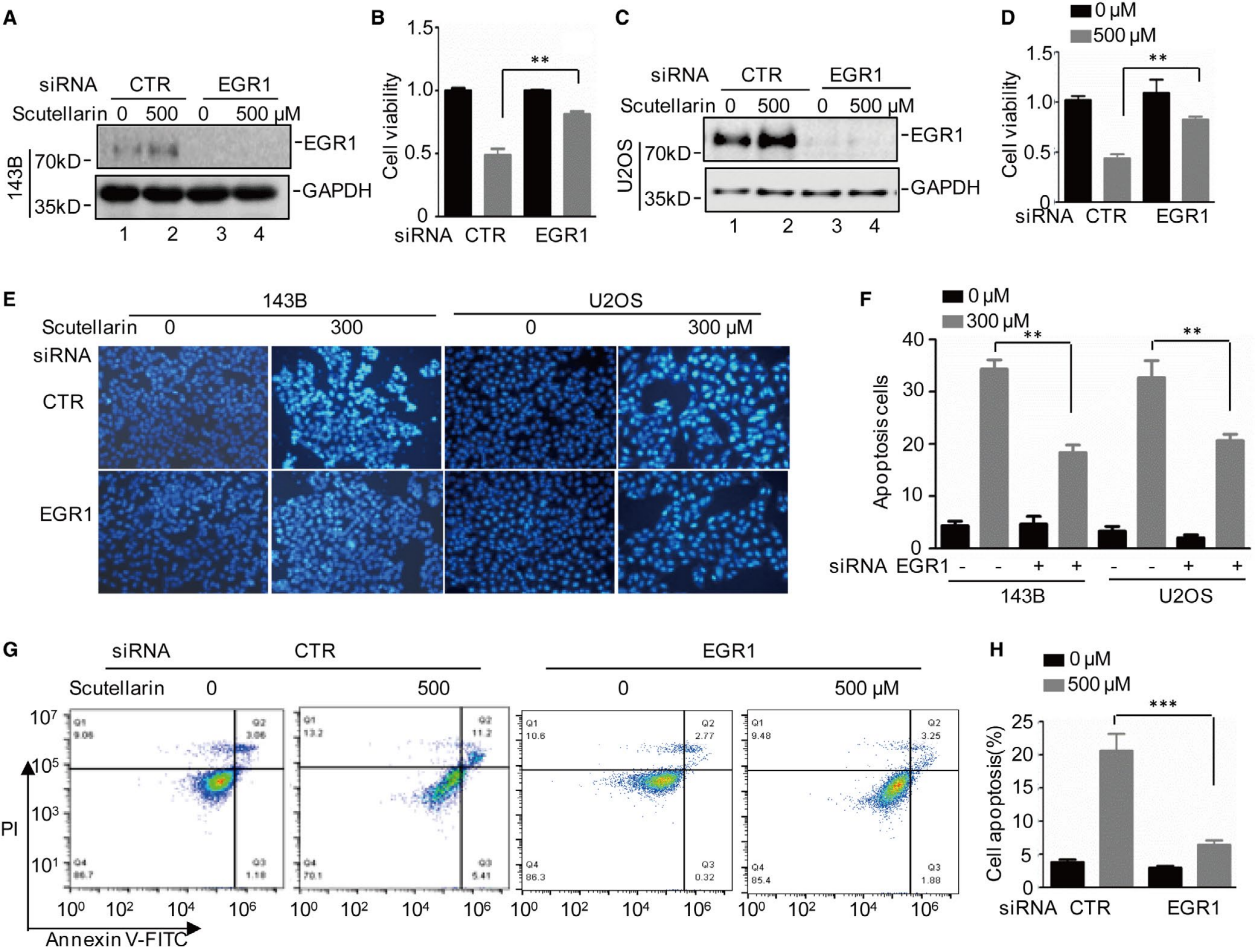
EGR1 contributes to anticancer effects of Scutellarin on osteosarcoma cells. (A‐D) EGR1 was knocked down in 143B and U2OS cells, and the cells were then treated with or without 500 μM Scutellarin. The EGR1 expression levels were analysed by Western blotting. Cell viability was detected by MTT assay. (E‐H) Cell apoptosis was analysed by Hoechst staining assay and flow cytometer. For (B), (D), (F) and (H), the results represent the mean ± SD of three independent experiments. **p < .01, ***p < .001 vs. control

To investigate the role of EGR1 in Scutellarin‐induced tumour suppression, we first used siRNA to knock down EGR1 in 143B and U2OS cells. Then, we treated these cells with or without 500 μM Scutellarin. The efficiency of knockdown was measured by Western blotting. We found that EGR1 was decreased when the cells were treated by EGR1 siRNA (Figure 3A,C). Then, cell viability was analysed by MTT, Hoechst 33342 staining and Annexin V/PI staining assays. We found that depletion of EGR1 abolished Scutellarin‐induced osteosarcoma cell viability decrease and cell apoptosis increase (Figure 3B,D and Figure 3E‐H). Collectively, these results indicate that EGR1 plays a pivotal role in anticancer effects of Scutellarin in osteosarcoma cells.

### 3.4 EGR1 transcriptionally suppresses LINC00857 expression in osteosarcoma cells

**FIGURE 4:**
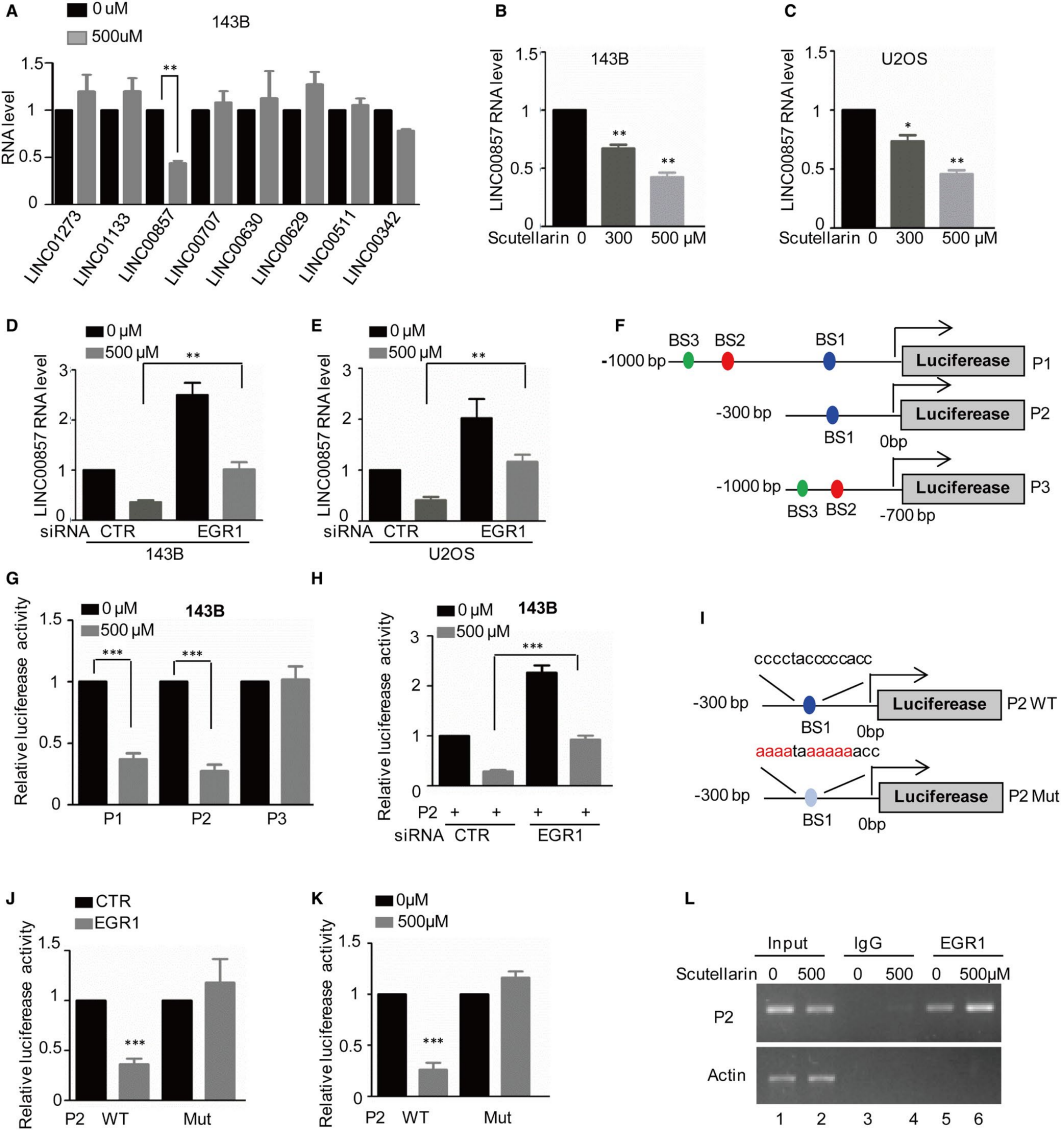
EGR1 transcriptionally suppresses LINC00857 expression in response to Scutellarin treatment. (A) 143B and U2OS cells were treated with or without 500 μM Scutellarin. The indicated LncRNAs were detected by qRT‐PCR. (B,C) 143B and U2OS cells were treated with Scutellarin as indicated. LINC00857 expression was analysed by qRT‐PCR. (D,E) EGR1 was knocked down in 143B and U2OS cells and the cells were then treated with or without 500 μM Scutellarin. LINC00857 expression was analysed by qRT‐PCR. (F) Schematic illustration of pGL3‐based reporter constructs, namely P1, P2 and P3, was used in luciferase assays to examine the potential EGR1 binding site. (G) P1, P2 and P3 were individually transfected into 143B cells with or without 500 μM Scutellarin treatment. Luciferase activity was measured. (H) P2 was transfected into 143B cells with or without EGR1 knockdown. Luciferase activity was measured. (I) Schematic illustration of EGR1 wild‐type binding site (BS1 WT) and the matching mutant (BS1 Mut) was used in luciferase assays. (J) The wild‐type binding site (BS1 WT) or the matching mutant (BS1 Mut) were individually transfected into 293T cells with or without EGR1 overexpression. Luciferase activity was measured. (K) BS1 WT and Mut were individually transfected into 143B cells. The cells were then treated with or without 500 μM Scutellarin. Luciferase activity was measured. (L) ChIP analysis showing the binding of EGR1 to LINC00857 in 143B cells with or without 500 μM Scutellarin treatment. An isotype‐matched IgG was used as a negative control. For (B), (C), (D), (E), (G), (H), (J) and (K), the results represent the mean ± SD of three independent experiments.*p < .05, **p < .01, ***p < .001 vs. control

Increasing studies have indicated that LncRNAs play important roles in cancer.^17^, ^18^ Therefore, we want to know whether LncRNAs are involved in Scutellarin‐induced tumour suppression. To this end, we first detected the expression of eight LncRNAs after Scutellarin treatment and found that Scutellarin significantly downregulated LINC00857 expression in 143B cells (Figure 4A). To further confirm this, we treated 143B and U2OS cells with or without 500 μM Scutellarin for 48 h and the expression of LINC00857 was measured by qRT‐PCR. As shown in Figure 4B,C, we found that LINC00857 was gradually downregulated following Scutellarin increase.

Combined with our previous results, we want to know whether EGR1 regulates LINC00857 expression in response to Scutellarin treatment. To prove it, we first knocked down EGR1 in 143B and U2OS cells and treated these cells with or without 500 μM Scutellarin for 48 h. Then, we detected the expression levels of LINC00857 by qRT‐PCR. We found that knockdown of EGR1 upregulated LINC00857 expression and reversed Scutellarin‐induced LINC00857 downregulation (Figure 4D,E).

To further prove whether EGR1 suppressed LINC00857 expression relying its transcriptional activity, we first inspected the upstream sequence of LINC00857 using the JASPAR software and identified three potential binding sites of EGR1 on LINC00857 promoter, which were named BS1, BS2 and BS3. To verify it, we cloned the promoter of LINC00857 and different truncations by PCR. We then inserted them into the pGL3‐based luciferase reporter plasmids, which were named P1‐P3 and transfected them into 143B cells with or without Scutellarin treatment (Figure 4F). As illustrated in Figure 4G, the luciferase activities of P1 and P2 were decreased in response to Scutellarin treatment. However, the decrease was abolished when P3 was transfected, suggesting that the region of P2 and BS1 was essential for Scutellarin‐induced LINC00857 downregulation. To further confirm that BS1 was indeed responsive to EGR1, we first transfected P2 into143B cells with or without EGR1 knockdown and luciferase activities were measured. As shown in Figure 4H, loss of EGR1 elevated the luciferase activities, which were suppressed by Scutellarin.

Then, the luciferase reporter plasmids containing BS1 WT and BS1Mut were constructed (Figure 4I). The BS1 WT and BS1 Mut were individually transfected into 293T cells as indicated and luciferase activities were measured. We found that the luciferase activity of BS1 WT but BS1 Mut was significantly decreased in response to EGR1 (Figure 4J). Similarly, we also obtained that Scutellarin dramatically inhibited the luciferase activity of BS1 WT but BS1 Mut in 143B cells (Figure 4K).

Subsequent ChIP assay showed that the chromatin fragment corresponding to the assumed EGR1 binding site (BS1) was specific in anti‐EGR1 immunoprecipitate of osteosarcoma cells, and the binding was enhanced after treatment with Scutellarin (Figure 4L). Taken together, these results demonstrate that EGR1 can bind to the promoter of LINC00857 and inhibit its expression in response to Scutellarin treatment.

### 3.5 LINC00857 contributes to Scutellarin‐induced c‐Myc downregulation via acting as a miR‐150‐5p sponge

**FIGURE 5:**
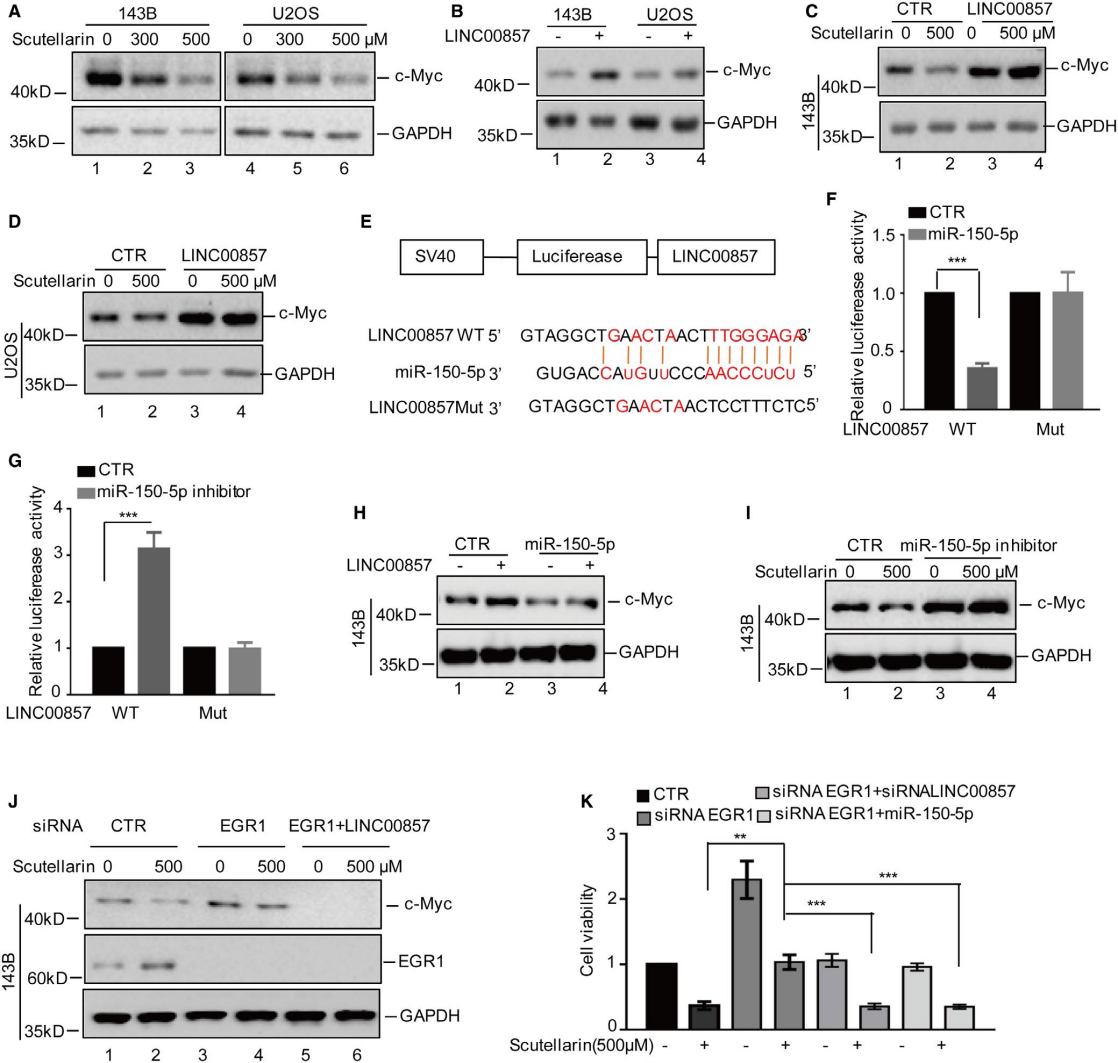
LINC00857 contributes to Scutellarin‐induced c‐Myc downregulation via acting as a miR‐150‐5p sponge. (A) 143B and U2OS cells were treated with Scutellarin as indicated. The expression levels of c‐Myc were analysed by Western blotting. (B) LINC00857 was overexpressed in 143B and U2OS cells. The protein levels of c‐Myc were detected by Western blotting. (C,D) 143B and U2OS cells with or without LINC00857 overexpression were treated with or without 500 μM Scutellarin, and the expression levels of c‐Myc were analysed by Western blotting. (E) Schematic diagrams of luciferase reporters containing the wild‐type binding site of miR‐150‐5p on LINC00857, named LINC00857 wild type (LINC00857 WT), and the mutant binding site, named LINC00857 Mut. (F,G) LINC00857 WT or Mut together with the miR‐150‐5p mimics or inhibitor was transfected into 143B cells. Luciferase activity was measured. (H) miR‐150‐5p mimics were transfected into 143B cells with or without LINC00857 overexpression, and the expression levels of c‐Myc were analysed by Western blotting. (I) miR‐105‐5p inhibitor was transfected into 143B cells with or without 500 μM Scutellarin. Then, the expression levels of c‐Myc were analysed by Western blotting. (J) LINC00857 siRNAs were transfected into 143B cells with or without EGR1 depletion, and the cells were treated with or without 500 μM Scutellarin. The expression levels of c‐Myc and EGR1 were analysed by Western blotting. (K) LINC00857 siRNAs or miR‐150‐5p mimics were transfected into 143B cells with or without EGR1 depletion, and the cells were treated with or without 500 μM Scutellarin. The cell viabilities were measured by MTT assay. For (F), (G) and (K), the results represent the mean ± SD of three independent experiments. **p < .01, ***p < .001 vs. control

Previous studies have indicated that the depletion of LINC00857 suppresses cell proliferation and induces cell apoptosis via decreasing some oncogenic proteins including c‐Myc in oesophageal adenocarcinoma.^19^ Thus, we ask whether LINC00857 contributes to Scutellarin‐induced c‐Myc downregulation in osteosarcoma cells. To this end, we first detected protein levels of c‐Myc in response to Scutellarin treatment. As shown in Figure 5A, we found that Scutellarin significantly inhibited c‐Myc expression in osteosarcoma cells. Then, we assessed the effect of LINC00857 on c‐Myc upon Scutellarin treatment. We found that overexpression of LINC00857 increased c‐Myc expression and reverse the decrease of c‐Myc induced by Scutellarin in 143B and U2OS cells (Figure 5B‐D).

Increasing studies have indicated that LINC00857 acts as a miRNAs sponge to regulate tumour progression.^20^, ^21^ Thus, we hypothesize that LINC00857 contributes to Scutellarin‐induced c‐Myc downregulation by binding miRNAs. To test this, we first predicated the binding miRNAs of LINC00857 from the miRDB and found that LINC00857 contained a potential binding region of miR‐150‐5p, which was reported to target c‐Myc and inhibited c‐Myc expression^22^ (Figure 5E).

To further verify the relationship between LINC00857 and miR‐150‐5p, we first inserted the wild‐type LINC00857 or the mutant of the binding site in the reporter plasmid (Figure 5E). We then transfected them into 143B cells with or without miR‐150‐5p mimics treatment and found that miR‐150‐5p significantly decreased luciferase activity of wild‐type LINC00857 (Figure 5F). However, the decrease of luciferase activity was abolished when the LINC00857 was mutated. Consistently, miR‐150‐5p inhibitor increased luciferase activity of wild‐type LINC00857 but the mutant (Figure 5G).

To further determine whether LINC00857 acted as a miR‐150‐5p sponge to promote c‐Myc expression, we transfected miR‐150‐5p into 143B cells with or without LINC00857 overexpression. Then, c‐Myc expression was detected by Western blotting. As shown in Figure 5H, the increase in c‐Myc protein levels by LINC00857 was disappeared when miR‐150‐5p was overexpressed. Subsequently, miR‐150‐5p inhibitor was introduced into 143B cells, and then, the cells were treated with or without Scutellarin treatment. We found that downregulation of c‐Myc induced by Scutellarin treatment was reversed by miR‐150‐5p inhibitor (Figure 5I).

Moreover, to investigate whether EGR1 facilitated anticancer effect of Scutellarin on osteosarcoma cell through regulating LINC00857/miR‐150‐5p/c‐Myc axis, we first inhibited EGR1 in 143B cells with or without LINC00857 knockdown, and then, these cells were treated with or without 500 μM Scutellarin for 48 h. The expression of c‐Myc was detected by Western blotting. We found that the loss of EGR1 abolished Scutellarin‐induced c‐Myc downregulation and the phenotype was reversed by LINC00857 inhibition (Figure 5J). Consistently, we found that knockdown of EGR1 prevented Scutellarin‐induced cell viability decrease, and the phenotype was reversed by LINC00857 inhibition or miR‐150‐5p overexpression (Figure 5K). These data indicate that LINC00857 contributes to Scutellarin‐induced c‐Myc downregulation via acting as a miR‐150‐5p sponge and EGR1 promotes anticancer effect of Scutellarin on osteosarcoma cells through regulating LINC00857/miR‐150‐5p/c‐Myc axis.

## 4 DISCUSSION

Scutellarin is an active flavone extracted from *Erigeron breviscapus*. Many reports have shown that Scutellarin exerts anticancer actions by multiple mechanisms in cancer.^23^ However, the antitumour effect and mechanism of Scutellarin on osteosarcoma cells remain unclear. In this study, we found that Scutellarin inhibited the proliferation and induced apoptosis of osteosarcoma cells by upregulating the expression of EGR1. Elevated EGR1 can directly bind to LINC00857, suppressing its transcription in response to Scutellarin treatment. Subsequently, we found that Scutellarin decreased c‐Myc expression via LINC00857 downregulation. Our data also revealed that LINC00857 acted as a miR‐150‐5p sponge to participate in Scutellarin‐induced c‐Myc downregulation. Taken together, these results suggest that exhibits antitumour effects in osteosarcoma.

Osteosarcoma is a dominating malignant bone tumour with high mortality. Owning to the cancer cell erosion and surgery resection, osteosarcoma always causes bone defects, resulted in inevitable dysfunction and disfigurement.^24^, ^25^, ^26^ Recently, the prevailing treatments for osteosarcoma are resection, neoadjuvant and adjuvant chemotherapy. However, anatomical constraints and resistance to conventional chemotherapies are often a barrier to osteosarcoma treatment. In this study, we found that Scutellarin is a potential therapeutic drug for osteosarcoma, which inhibited osteosarcoma cell growth and promoted cell apoptosis in vivo and in vitro. Our data indicated that Scutellarin may be beneficial to decrease tumour size and clinical nursing of patients with osteosarcoma.

A number of studies have shown that many lncRNAs are involved in the occurrence and development of various cancers.^14^, ^27^ At the same time, many transcription factors are involved in regulating lncRNAs in cancer. For example, FoxO1 facilitated transcription of GAS5 to promote propofol‐induced oral squamous cell carcinoma apoptosis.^28^ EGR1 belongs to the EGR family and has been implicated in the regulation of cancer cell growth, metastasis and apoptosis.^29^, ^30^, ^31^ An increasing amount of evidences have shown that EGR1 is involved in osteosarcoma development and progression, particularly as a tumour suppressor.^32^

Here, we demonstrated that the protein and mRNA levels of EGR1 were increased under Scutellarin treatment. Increased EGR1 transcriptionally suppressed LINC00857 expression in response to Scutellarin treatment. However, the molecular mechanism of EGR1 increase induced by Scutellarin is still unknown and we will uncover it in the future.

Accumulating studies have revealed that c‐Myc has been characterized as a vital oncogene, which is correlated with cell proliferation, apoptosis and metastasis in various human cancers. In human hepatocellular carcinoma, the downregulation of c‐Myc inhibited tumour proliferation.^33^ In osteosarcoma, c‐Myc has been shown to promote the growth of tumour cellsss.^34^ Overall, we found that protein levels of c‐Myc were decreased in response to Scutellarin treatment in osteosarcoma cells, which relied on the LINC00857 decrease by Scutellarin. LINC00857 was reported to act as an oncogene in many cancers including hepatocellular carcinoma, lung cancer, pancreatic cancer and gastric cancer.^15^, ^20^, ^35^, ^36^ Many studies have suggested that LINC00857 can function as an miRNAs sponge to regulate signalling pathways and biological functions.^21^ Consistently, our study revealed that LINC00857 contributes to Scutellarin‐induced c‐Myc downregulation via acting as a miR‐150‐5p sponge. Moreover, we found that miR‐150‐5p or c‐Myc knockdown eliminated increases in LINC00857‐induced cell viability under Scutellarin treatment. Thus, our data suggest that EGR1/LINC00857/miR‐105‐5p/c‐Myc axis plays an important role in Scutellarin‐inhibited osteosarcoma cell growth.

## CONFLICT OF INTEREST

The authors declare no competing interests.

## AUTHOR CONTRIBUTIONS

**Jian Han:** Data curation (equal); Formal analysis (equal); Writing‐original draft (equal). **Peng Wang:** Data curation (equal); Validation (equal). **Xin Xia:** Investigation (equal); Writing‐review & editing (equal). **Li Zhang:** Data curation (equal); Validation (equal). **He Zhang:** Formal analysis (equal). **Yu Huang:** Data curation (equal). **Xiaodong Li:** Formal analysis (equal); Writing‐original draft (equal). **Wenzhi Zhao:** Funding acquisition (equal); Writing‐original draft (equal); Writing‐review & editing (equal). **Lu Zhang:** Writing‐original draft (equal).

## Data Availability

Data will be made available on request to the corresponding authors.
